# Association between urolithiasis and metabolic syndrome in the Aksu region of Xinjiang, China: A cross-sectional study

**DOI:** 10.1097/MD.0000000000045862

**Published:** 2025-11-07

**Authors:** Ge Zhang, Chang Liu, Zhipeng Liu, Qiang Yao, Renxiong Cao, Chuanjiang Ling

**Affiliations:** a Department of Nephrology, The Second People’s Hospital of Aksu Prefecture, Aksu, Xinjiang Province, China; b Department of Urology, The Second People’s Hospital of Aksu Prefecture, Aksu City, Xinjiang Province, China; c Department of Information Network, The Second People’s Hospital of Aksu Prefecture, Aksu City, Xinjiang Province, China.

**Keywords:** hyperglycemia, hypertension, metabolic syndrome, urolithiasis

## Abstract

This study explores the association between adult urolithiasis and metabolic syndrome (MetS) in Aksu region of Xinjiang. In the context of this cross-sectional analysis, we included 11,208 hospitalized patients aged 18 to 80 years from our hospital between June 1, 2023, and June 31, 2024. Urolithiasis was diagnosed according to the 2022 edition of the “Guidelines for the Diagnosis and Treatment of Urological Diseases in China,” the diagnostic criteria for MetS were based on the 2004 Chinese Diabetes Society standards. Multivariable logistic regression was used to examine the associations between MetS and urolithiasis. The prevalence of urolithiasis in this region was higher in males than in females (3.7% vs 1.5%, *P* < .01), and the Han Chinese participants had a higher prevalence (4.5% vs 1.7%, *P* < .01), of urolithiasis than other ethnic group participants. In all age groups, the peak prevalence was between the ages of 30 and 40 years (3.63%). MetS independently increased the likelihood of urolithiasis, with a 1.461 times higher prevalence rate in those with MetS (95% confidence interval: 1.060–2.013) than in those without MetS. Among the MetS components, hyperglycemia and hypertension were significant influencing factors for urolithiasis in this population. In male patients, lipid-related metabolic indicators also had a significant impact on the occurrence of urolithiasis. Metabolic syndrome or metabolic related indexes such as blood sugar, triglyceride, and diastolic blood pressure have significant influence on the occurrence of urolithiasis in Han patients. Urolithiasis was more common in Han Chinese males in this region, with a trend toward younger age. Metabolic syndrome was an important risk factor for urolithiasis. Our study suggests that those with MetS require vigilant monitoring for urolithiasis, and clinical procedures should incorporate screening for it.

## 1. Introduction

Urolithiasis is characterized by the formation of stones in the kidneys, bladder, and ureters, with kidney and ureteral stones being the most common. Urolithiasis is a common disease of the urinary system. It is a widespread condition with high morbidity worldwide. Owing to the bad dietary habits, lack of exercise patterns, and improving of diagnostic methods, its incidence has been gradually increasing. The overall prevalence is approximately 5% to 9% within Europe, 13% in North America, and 5.8% in China.^[1,2]^ Over the past 40 years, the incidence of urolithiasis has increased globally.^[3]^ China, which is among the top 3 regions with the highest rates of urolithiasis, also exhibits notable differences in urolithiasis prevalence across its regions.^[4]^

If not managed promptly, urolithiasis can lead to complications such as urinary tract obstruction, hydronephrosis, infection, sepsis, and even end-stage renal disease, with the recurrence rate over 5 years can reach up to 50%,^[5,6]^ resulting in a significant medical and social health burden. According to statistics, there are 106 million cases of urolithiasis globally in 2021, causing a significant disease burden and increasing the risk of death and disability.^[7]^ These findings indicate the importance of identifying factors influencing urolithiasis formation, to prevent initial stone formation and recurrence, which are of clinical importance.

The causes of urolithiasis are complicated and have been reported to be closely related to genetic factor, eating habits, lifestyle, and environmental factor.^[4]^ Recent studies have demonstrated that metabolic syndrome (MetS) is a significant risk factor for urolithiasis.^[8,9]^ It has been reported that the incidence of urolithiasis in patients with obesity and MetS is significantly higher than in individuals of normal weight.^[10]^ Most patients with urinary calculi have different types and degrees of metabolic abnormalities.^[11]^ Since the incidence of urolithiasis varies significantly in different regions, ongoing research into the factors (especially MetS) contributing to urolithiasis in specific regions is essential for reducing its incidence and recurrence.

Aksu Prefecture in the Xinjiang Uygur Autonomous Region, which is located in the southern arid area of northwest China and has 22.89% Han Chinese and 77.11% other ethnic populations, has unique geographical environments and dietary habits compared with those in other parts of northwest China.^[12]^ Findings suggest that 32.85% of the Han and Uygur populations in Xinjiang suffer from MetS.^[13]^ Currently, the risk factors of urolithiasis in this region are still unclear, especially the correlation between the occurrence of urolithiasis and MetS in this region needs to be clarified. So in the present study, we selected inpatients from a local hospital from June 1, 2023 to June 30, 2024. Then we conducted a retrospective study to explore the metabolic differences between urolithiasis patients and non-urolithiasis patients regarding baseline data, serum biochemistry, and urine examination. It aims to identify risk factors for urolithiasis formation, offering insights for a comprehensive approach for managing and preventing the condition, highlighting key differences and potential preventive strategies.

## 2. Methods

### 2.1. Study participants and grouping

**Figure 1.**
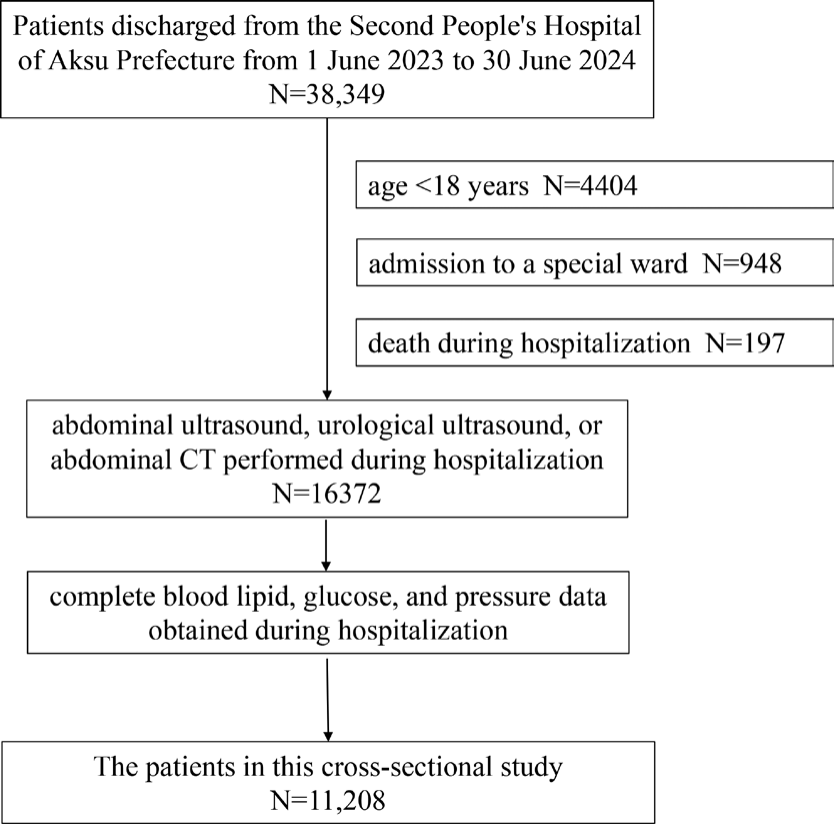
The process of screening clinical data.

In this study with a cross-sectional design, 11,208 participants were all hospitalized patients between June 1, 2023, and June 30, 2024 (the screening process is shown in Fig. 1). The inclusion criteria were as follows: abdominal ultrasound, urological ultrasound, or abdominal computed tomography performed during hospitalization, age ≥ 18 years, and complete blood lipid, blood glucose, and blood pressure data during hospitalization. The criteria for exclusion were as follows: age < 18 years; admission to the special ward; and death during hospitalization. Finally, a total of 11,208 patients were included.

Grouping: patients were categorized into 2 groups based on the presence of urinary calculi: the urolithiasis group and the non-urolithiasis group. The diagnostic criteria for urolithiasis were based on abdominal ultrasound, urological ultrasound, or abdominal computed tomography diagnoses of kidney, ureteral, or bladder stones.

### 2.2. Data collection and diagnosis methods

General information was obtained from the electronic medical record system of the hospital. Data from blood and urine laboratory tests were collected from the Hospital Information System and exported following guidelines set by the hospital’s information technology department. Ethical approval was obtained from the ethics committee of The Second People’s Hospital of Aksu Prefecture following the principles outlined in the Declaration of Helsinki. And exempted from informed consent (Ethics approval number: akseyyllsc-lw06).

The biochemical items of blood were serum potassium, sodium, chloride, triglycerides, total cholesterol, low-density lipoprotein cholesterol, high-density lipoprotein cholesterol (HDL-C), fasting blood glucose, indirect bilirubin, alanine transaminase, aspartate transaminase, total protein, albumin, globulin, blood urea nitrogen, blood creatinine, C-reactive protein, and blood uric acid, some routine urine indicators (urine pH, urine specific gravity, red blood cells, white blood cells, protein, and occult blood), and certain routine blood indicators (neutrophil count, lymphocyte count, monocyte count, and platelet count). The body mass index (BMI) was calculated as weight (kg)/height² (m²), and the triglyceride-glucose index (TyG) was calculated using the formula: TyG = ln[triglycerides (mg/dL) × fasting blood glucose (mg/dL)/2].

Metabolic syndrome was diagnosed based on the 2004 CDS criteria,^[14]^ which require the presence of 3 or more of the following components:

1. Overweight and/or obesity: BMI ≥ 25.0 kg/m².
2. Hyperglycemia: fasting plasma glucose ≥ 6.1 mmol/L (110 mg/dL) and/or 2-hour plasma glucose ≥ 7.8 mmol/L (140 mg/dL), and/or a diagnosis of diabetes with treatment.
3. Hypertension: blood pressure readings of 140/90 mm Hg or higher, or a confirmed diagnosis of hypertension with ongoing treatment.
4. Dyslipidemia: fasting triglyceride levels of at least 1.7 mmol/L (110 mg/dL), or fasting HDL-C levels below 0.9 mmol/L (35 mg/dL) for men.

### 2.3. Statistical analysis

The study data were analyzed and processed using SPSS software (version 26.0; IBM Corp., Armonk). Continuous variables are expressed as mean ± standard deviation ($\bar{x}\pm s$</mathgraphic) for normally distributed data and were compared using *t* tests. Non-normally distributed data are expressed as median (interquartile range) and were analyzed using the Mann–Whitney *U* test. Categorical variables are expressed as counts (percentages) and were analyzed using the chi-square test. Statistical significance was set at *P* < .05. Variables with *P* < .1 were included in the multivariable regression analysis, and binary logistic regression was used to analyze the relationship between MetS and other clinical features with urolithiasis.

## 3. Results

### 3.1. Demographic characteristics of study participants

This study included 11,208 participants, Table 1 summarizes the demographics and baseline characteristics. Of these 11, 208 participants, 5060 were male (45.15%) and 6147 were female (54.85%). Males experienced a higher rate of urolithiasis compared to females (3.7% vs 1.5%, *P* < .01). Among the ethnic groups, 3386 were Han Chinese (30.2%) and 7822 were from other ethnic groups (69.8%), with Uyghur patients accounting for 96.4% (n = 7522) of all patients. The incidence of urolithiasis was higher in the Han Chinese than in other ethnicities (4.5% vs 1.7%, *P* *<* .01). There were no significant age differences between the urolithiasis and non-urolithiasis groups (*P* > .05). Subgroup analysis with age showed that the incidence of urolithiasis was lower in the 18 to 30 years old group but increased with age, the highest incidence occurred in the group of 30 to 60 years old, then declined in the group of >60 years old. The incidence in the 30 to 40-year age group was significantly higher than that in the 18 to 30-year (3.63% vs 1.22%, *P* = .0032) and > 60-year age groups (3.63% vs 2.03%, *P* = .0029), but there was no significant difference compared with that in the 40 to 60-year age group (3.63% vs 2.94%, *P* = .2733).

**Table 1:** Demographic characteristics of the study participants.

	Urolithiasis (n = 277)	Non-urolithiasis (n = 10,931)	*P*-value
Sex			
Male	186 (3.7%)	4874 (96.3%)	<.001
Female	91 (1.5%)	6057 (98.5%)	
Ethnicity			
Han Chinese	146 (4.5%)	3240 (95.5%)	<.001
Other Ethnicities	131 (1.7%)	7691 (98.3%)	
Age (yr)	56.99 ± 14.31	58.15 ± 14.80	.198
[18–30]	8 (1.22%)	650 (98.78%)	<.001
(30–40]	33 (3.63%)	877 (96.37%)	
(40–60]	130 (2.94%)	4292 (97.06%)	
>60	106 (2.03%)	5112 (97.97%)	

In the age stratification, the mathematical collection of symbols is used to represent the range, “[]” means inclusion, “()” means exclusion.

### 3.2. Metabolic disorders characteristics of the urolithiasis and non-urolithiasis groups

The comparison results are shown in Table 2. Patients with hyperglycemia had a higher likelihood of developing urolithiasis compared to those without hyperglycemia. (3.0% vs 2.2%, *P* < .05). Hypertensive patients also exhibited a higher incidence of urolithiasis than non-hypertensive patients (2.8% vs 2.1%, *P* < .05). Compared to patients without urolithiasis, those with the condition had much higher levels of uric acid in their blood (*P* < .05). No significant differences in BMI were observed between the 2 groups (*P* > .05). There were also no significant differences in the incidence of urolithiasis between patients with and without dyslipidemia (*P* > .05). Moreover, a novel biomarker for evaluating insulin resistance, the fasting TyG index, was significantly higher in the urolithiasis group than in the non-urolithiasis group (*P* < .01). Considering the common metabolic disorders in patients from the Xinjiang region, we also analyzed these data according to the MetS CDS diagnostic criteria, finding that 3970 patients (35.4%) were diagnosed with MetS, patients with MetS had a notably higher rate of urolithiasis compared to those without it (64.6%) (3.0% vs 2.2%, *P* < .05, Table 2).

**Table 2:** Comparison of metabolic disorders between the urolithiasis and non-urolithiasis groups.

	Urolithiasis (n = 277)	Non-urolithiasis (n = 10,931)	*P*-value
BMI (kg/m²)	25.84 (23.45, 28.81)	25.71 (22.98, 28.65)	.522
Hyperglycemia			
Yes	102 (3%)	3294 (97%)	.017
No	175 (2.2%)	7637 (97.8%)	
Hypertension			
Yes	161 (2.8%)	5510 (97.2%)	.011
No	116 (2.1%)	5421 (97.9%)	
Dyslipidemia			
Yes	210 (2.5%)	8152 (97.5%)	.641
No	67 (2.4%)	2779 (97.6%)	
Metabolic syndrome			
Yes	119 (3.0%)	3851 (97.0%)	.008
No	158 (2.2%)	7080 (97.8%)	
TyG index	7.31 (6.97, 7.82)	7.16 (6.78, 7.63)	<.001
UA (μmol/L)	328.60 (262.23, 406.30)	277.01 (221.00, 340.28)	<.001

BMI = body mass index, TyG = fasting triglyceride-glucose index, UA = uric acid.

As the prevalence of urolithiasis was significantly higher in men than in women, we further stratified the study participants by sex to observe differences in metabolic indicators, such as BMI, between the urolithiasis and non-urolithiasis groups. The results showed that among male patients, the urolithiasis group had significantly higher BMI, HDL-C level, total cholesterol level, and TyG index than the non-urolithiasis group (*P* < .05, Table 3), while among female patients, no significant differences were observed in these indicators between the urolithiasis and non-urolithiasis groups (*P* > .05, Table 3).

**Table 3:** Comparison of metabolic indicators between the urolithiasis and non-urolithiasis groups by sex.

	Male (n = 5060)			Female (n = 6148)		
	Urolithiasis (n = 186)	Non-urolithiasis	*P*-value	Urolithiasis (n = 91)	Non-urolithiasis	*P*-value
BMI	26.15 (23.77, 29.38)	25.71 (23.15, 28.41)	.041	25.00 (22.68, 27.73)	25.71 (22.80, 28.76)	.204
HDL-C	0.92 (0.79, 1.05)	0.85 (0.74, 1.00)	.022	0.97 (0.80, 1.19)	0.95 (0.80, 1.15)	.535
TC	4.08 (3.51, 4.49)	3.81 (3.22, 4.53)	<.001	4.20 (3.57, 5.28)	4.20 (3.55, 4.94)	.312
TyG	7.35 (6.90, 7.83)	7.10 (6.72, 7.57)	.<001	7.21 (6.99, 7.11)	7.21 (6.83, 7.68)	.108

BMI = body mass index, HDL-C = high-density lipoprotein cholesterol, TC = total cholesterol, TyG = triglyceride-glucose index.

Compared with gender stratification, ethnic stratification seems to have more research value. We stratified the subjects by ethnicity, and observed the differences of indexes between the urolithiasis and non-urolithiasis group. The results showed that among Han patients the incidence of urinary calculi in patients with MetS was higher than that in patients with non-MetS (5.6% vs 3.6%, *P* < .05, Table 4), and there was no significant difference among patients of other nationalities in China. In Han patients, metabolic related indexes such as hyperglycemia, diastolic blood pressure, triglyceride, and TyG index are significantly higher than those in patients with non-urinary calculi (*P* < .05, Table 4), while in other nationalities in China, there is no significant difference between urinary calculi group and non-Nationalities groups (*P* > .05, Table 4).

**Table 4:** Comparison of Indicators between the Urolithiasis and Non-Urolithiasis Groups by Nationalities in China.

	Han Chinese (n = 3386)			Other Nationalities in China (n = 7822)		
	Urolithiasis (n = 146)	Non-Nationalities	*P*-value	Urolithiasis (n = 131)	Non-Nationalities	*P*-value
Sex						
Male	99 (5.7%)	3240 (94.3%)	<.001	87 (2.6%)	3227 (97.4%)	<.001
Female	47 (2.9%)	1593 (97.1%)		44 (1.0%)	4464 (99.0%)	
Hyperglycemia						
Yes	65 (5.4%)	1149 (94.6%)	0.026	37 (1.7%)	2145 (98.3%)	.929
No	81 (3.7%)	2091 (96.3%)		94 (1.7%)	5546 (98.3%)	
Metabolic syndrome						
Yes	65 (5.6%)	1100 (94.4%)	0.009	54 (1.9%)	2751 (98.1%)	.197
No	81 (3.6%)	2140 (96.4%)		77 (1.5%)	4940 (98.5%)	
TG (mmol/L)	1.92	1.64	<.001	1.37	1.39	.907
	(1.37, 2.64)	(1.17, 2.52)		(1.08, 1.92)	(1.01, 2.02)	
D-BP (mm Hg)	82	79	<.001	82	79	.042
	(73, 90)	(71, 88)		(72, 90)	(71, 88)	
Scr (μmol/L)	78.0	69.0	<.001	70.0	64.0	<.001
	(66.2, 96.0)	(57.0,81.9)		(56.0,89.0)	(54.0,76.0)	
BUN (mmol/L)	5.21	4.88	0.02	4.91	4.73	.102
	(4.15,6.48)	(3.38,6.15)		(4.01,6.24)	(3.76,5.92)	
UA (μmol/L)	337.46	306.60	<.001	314.4	264.84	<.001
	(280.30,416.64)	(250.91,370.18)		(239.48,396.60)	(212.89,324.46)	
TyG	7.52	7.31	0.005	7.14	7.10	.629
	(7.05, 7.90)	(6.92, 7.84)		(6.76, 7.56)	(6.73, 7.55)	

BUN = blood urea nitrogen, D-BP = diastolic blood pressure, Scr = serum creatinine, TyG = triglyceride-glucose index, UA = uric acid.

### 3.3. Serum and urine biochemical characteristics of the urolithiasis and non-urolithiasis groups

The comparison results are shown in Table 5. Blood sample results showed that patients with urolithiasis had significantly higher levels of indirect bilirubin, total protein, albumin, blood urea nitrogen, and serum creatinine than those without urolithiasis (*P* < .05). Among them, the difference between ALB and Scr is the most significant. There was no significant difference in globulin level between the 2 groups (*P* > .05).

**Table 5:** Comparison of biochemical indicators between the urolithiasis and non-urolithiasis groups.

	Urolithiasis (n = 277)	Non-urolithiasis (n = 10,931)	*P*-value
Blood test index			
I-Bil (mmol/L)	6.79 (4.59, 9.82)	6.23 (4.24, 9.02)	.007
TP (mmol/L)	69 (65, 73)	68 (64, 72)	.009
ALB (mmol/L)	40 (36, 43)	39 (35, 42)	<.001
GLO (mmol/L)	29 (26, 33)	29 (26, 33)	.317
BUN (mmol/L)	5.07 (4.15, 6.44)	4.90 (3.96, 6.10)	.001
Scr (μmol/L)	76.00 (64.00, 93.00)	67.00 (56.95, 79.00)	<.001
CRP	17.87 (10.25, 37.83)	19.31 (10.37, 36.37)	.114
NEU (×10^9^/L)	4.40 (3.31, 5.68)	4.17 (3.23, 5.51)	.039
Urinalysis index			
Urine pH	6.00 (6.00, 6.50)	6.00 (6.00, 6.50)	.396
Urine protein			
Positive	47 (4.1%)	1092 (95.9%)	<.001
Negative	206 (2.4%)	8434 (97.6%)	
Urine leukocytes			
Positive	74 (3.5%)	2059 (96.5%)	.004
Negative	179 (2.3%)	7467 (97.7%)	
Urine occult blood			
Positive	76 (4.9%)	1467 (95.1%)	<.001
Negative	117 (2.1%)	8059 (97.9%)	
Urine nitrite			
Positive	20 (3.9%)	498 (96.1%)	.061
Negative	233 (2.5%)	9028 (97.5%)	

ALB = albumin, BUN = blood urea nitrogen, CRP = C-reactive protein, GLO = globulin, I-Bil = indirect bilirubin, NEU = neutrophil count, SCr = serum creatinine, TP = total protein.

Additionally, the incidence of urolithiasis was higher among patients with positive urine protein, leukocytes, and occult blood than among those with negative results (4.1% vs 2.4%, *P* < .05; 3.6% vs 2.3%, *P* < .05; 4.9% vs 2.1%, *P* < .05, respectively, Table 5). The incidence of urolithiasis showed no significant difference between patients with positive and negative urinary nitrite results (*P* > .05, Table 5). Further, there were no significant differences between the urolithiasis and non-urolithiasis groups in terms of urine pH and specific gravity (*P* > .05, Table 5).

### 3.4. Multivariate analysis of factors influencing the occurrence of urolithiasis

To further explore factors influencing urolithiasis, we performed a multivariate analysis using urolithiasis as the dependent variable and the factors with *P* < .1 from the above results (sex, ethnicity, MetS, indirect bilirubin, total protein, albumin, urea nitrogen, creatinine, uric acid, neutrophil count, and TyG index) as independent variables in binary logistic regression (Table 6) analysis. The results showed that Han ethnicity and male sex were associated with a higher probability of urolithiasis [odds ratio [OR] = 2.313 (1.672, 3.199) and OR = 1.846 (1.291, 2.640) *P* < .01] and patients with MetS had a higher risk of urolithiasis than those without MetS [OR = 1.461 (1.060, 2.013), *P* < .05]. Moreover, the urolithiasis group showed significantly higher neutrophil counts and uric acid levels than the non-urolithiasis group (*P* < .01).

**Table 6:** Results of the binary logistic regression analysis.

Characteristic	β	SE	*P*	OR	OR (95% CI)	
					Lower limit	Upper limit
Ethnicity	0.838	0.166	<.001	2.313	1.672	3.199
Sex	0.613	0.183	.001	1.846	1.291	2.640
Metabolic syndrome	0.379	0.164	.021	1.461	1.060	2.013
Neutrophil count	0.110	0.031	<.001	1.116	1.050	1.186
Uric acid	0.003	0.001	<.001	1.003	1.002	1.004

CI = confidence interval, OR = odds ratio.

## 4. Discussion

This study mainly investigated the hospital admission records of patients over the past year at a tertiary hospital, revealing a trend toward younger ages among patients with urolithiasis. Additionally, patients with MetS were found to have a significantly higher proportion of urolithiasis than those without MetS. Among the components of MetS, hyperglycemia and hypertension were identified as the major influencing factors for urolithiasis in this region. Moreover, ethnic and sex factors were found to significantly affect the occurrence of urolithiasis, with a notable association between increased blood lipid metabolic indicators and urolithiasis in male patients.

The study results indicate that the different ethnic and sex of patients are associated with the incidence of urinary stones Han ethnic patients exhibited a higher prevalence of urolithiasis than other ethnic groups, which may be related to population genetic differences or lifestyle. For example, the incidence of kidney stones differs among Black and White populations, possibly due to differences in 24-hour urine volume and urinary calcium excretion, with Black patients having significantly lower levels than White patients, as shown in a retrospective cohort study from Chicago, USA.^[15]^ This may be related to high-protein diet, moreover which is an evolutionary benefit in view of Africa’s climate and the need to save water.

The varying incidence rates of urolithiasis among different genetic groups may generate phenotypic differences. A study analyzing urinary regulatory proteins (uromodulin [UMOD]) in a multiethnic biobank and comparing the clinical codes for kidney stones in the Million Veteran Program found that variations in UMOD/PDILT genes are associated with kidney stone incidence across different races.^[16]^ Common variants in the UMOD gene are considered an evolutionary adaptation and have been implicated in kidney stone formation. Therefore, we suspect that eating habits have caused differences in urinary calculi among ethnic groups, but the indigenous peoples in this area have gained evolutionary protection advantages. At present, no one has studied whether different ethnic groups in China are closely related to the occurrence of urinary calculi. However, according to our research data, compared with other ethnic minorities, the Han nationality is more prone to urinary calculi. Metabolic related factors are closely related to urinary calculi in Han patients, but there is no difference in the performance of other ethnic minorities. We speculate that this may be related to the genetic protective changes caused by long-term diet or living conditions, and this speculation needs further study.

The present study also found that the probability of developing urolithiasis was significantly higher in male patients than in female patients, a result consistently observed in many countries and regions. A retrospective study by Sáenz Medina on 106,407 hospitalized patients with kidney or ureteral stones in Spain reported similar results.^[17]^ Rule et al also reported that male gender, age, and ethnicity are important risk factors for kidney stones.^[18,19]^ In a study by Peng et al, male mice in an acetaldehyde-induced kidney stone model showed more calcium salt crystals in the renal medulla than female and castrated male mice, suggesting that testosterone promotes urolithiasis.^[20]^ Other studies have shown that inhibiting the expression of androgen receptors in renal tubular cells can significantly reduce the deposition of calcium salt crystals in the kidneys of mice and rats induced by acetaldehyde.^[21]^ Zhu et al also revealed that knock-out of estrogen receptor β or use of an estrogen receptor β antagonist can lead to a significant increase in kidney stone formation in female mice induced by acetaldehyde.^[22]^ Thus, we speculate that the higher incidence of urolithiasis in males may be related to hormone levels or hormone receptors.

This present study also found that the most common age for urolithiasis was between 30 and 40 years old. A global systematic analysis of the disease burden of urolithiasis from 2000 to 2021 showed that the age period of highest incidence is 55 to 59 years old.^[8]^ However, the results of our study suggest a trend of younger onset in recent years, with the age range shifting to 30 to 40 years old, which could be related to the lifestyle changes and the living geographical features of Akesu region.

With changes in lifestyle, there has been an increasing intake of carbohydrates and fats and a decreasing in physical activity, leading to various metabolic abnormalities, such as obesity, diabetes, hypertension, and hyperlipidemia, even being a new risk factor of urolithiasis. A cohort study from Korea found that elevated blood glucose levels and insulin resistance significantly increased the incidence of kidney stones.^[23]^ Another large cohort study on urolithiasis found that compared to patients with normal blood pressure, those with hypertension and urolithiasis had significantly lower levels of citrate in their 24-hour urine, suggesting that lower citrate levels in hypertensive patients may promote the formation of urolithiasis.^[24]^ Our study confirmed that the patients with hyperglycemia or hypertension had a high prevalence of urolithiasis than those without hyperglycemia or hypertension. Although our study did not directly measure insulin levels, we assessed the TyG index, a method used to evaluate insulin resistance and cardiovascular metabolic risk.^[25]^ Our results revealed that the TyG index was significantly higher in patients with urolithiasis than in those without urolithiasis, especially among male patients. Zheng et al conducted a cross-sectional study of 84,968 individuals and found that the TyG index showed a nonlinear dose–response relationship with the risk of kidney stones in men,^[26]^ which is consistent with our findings.

Given the possibility of multiple metabolic disorders in patients, we further analyzed whether MetS is related to the occurrence of urolithiasis, and the results indicated that patients with MetS had a significantly higher risk of developing urolithiasis than those without MetS. This result is similar to the meta-analysis of MetS reported by Dassanayake et al.^[8]^ Clinical research by Prochaska et al also found that patients with kidney stones had higher fasting blood glucose levels and were more prone to insulin resistance than healthy individuals.^[27]^ This suggests that urolithiasis may be causally related to MetS.

After excluding confounding factors, we performed a multivariate statistical analysis and found that, besides the sex and ethnicity, MetS was the most significant risk factor for the occurrence of urolithiasis, with patients with MetS having a significantly higher risk (OR value of 1.461). A prospective cohort study by Liu et al on 487,860 participants from the UK Biobank also suggested that MetS is an important independent risk factor for kidney stone development in European populations.^[27]^ A previous study used a high-fat diet to successfully induce MetS in male rats and found that, compared to lean rats, rats with MetS more easily developed calcium oxalate kidney stones when induced by ethylene glycol and ammonium chloride. The study also found that the increased risk of kidney stones in rats with MetS was associated with elevated uric acid, inflammation, and reduced antioxidant levels.^[28]^ Our study also found that uric acid levels were higher in patients with urolithiasis than in those without urolithiasis, indicating that patients with urolithiasis may have purine metabolism abnormalities.

Furthermore, our study revealed that neutrophil levels were higher in patients with urolithiasis than in those without urolithiasis. Compared to other white blood cell types, neutrophils are more sensitive to early inflammatory responses and are considered inflammatory biomarkers, particularly in patients with MetS.^[29,30]^ Research indicates that when neutrophil levels increase, neutrophils adhere to endothelial cells and cause endothelial damage through reactive oxygen species and tumor necrosis factor, accelerating kidney damage progression.^[31]^ Sfoungaristos et al conducted a clinical study on 156 kidney stone patients and found that neutrophil counts could reflect the early inflammatory response caused by urolithiasis in kidney tissue and ureteral mucosa and could be used as a potential indicator for predicting spontaneous stone passage in renal colic patients.^[32]^ Our multivariate analysis also showed that neutrophils and uric acid were significant risk factors for urolithiasis.

Our study found the prevalence of urolithiasis had no significant difference between patients with and without hyperlipidemia, when we analyzed the data by sex, we found that male patients with hyperlipidemia had a significantly higher risk of developing urolithiasis than those without hyperlipidemia. This phenomenon was not observed in female patients. Some studies have suggested that elevated blood lipids increase fatty acid concentrations, which further lowers urine pH, leading to highly acidic urine and reducing the saturation of uric acid and calcium oxalate compounds in the urine, thereby promoting the formation of uric acid stones.^[33]^ A large cohort study of the Taiwan Biobank found that high triglyceride, low HDL-C, and a high total cholesterol-to-HDL-C ratio were associated with an increased incidence of urolithiasis.^[34]^ Our findings do not entirely align with these results, which may be attributed to differences in ethnic composition and local lifestyles; further research is needed to explore these factors.

Although our study found a significant correlation between positive urine occult blood, protein, and white blood cells and the risk of developing urolithiasis, multivariate analysis showed that these factors were not strongly related to the occurrence of urolithiasis. This study has certain advantages, as the data were derived from hospitalized patient information, which supports the validity of the research findings. At the same time, because all the participants are from hospitals and suffer from various diseases, this study only controls metabolic diseases such as diabetes, hypertension, obesity, hyperlipidemia, and hyperuricemia as confounding factors, and does not intervene other diseases, so the research results may have certain limitations. Since this was a cross-sectional study, further prospective research is needed to explore the relationship between these factors and the occurrence of urolithiasis.

## 5. Conclusion

The present study indicates that Han people, male sex, young age, and MetS are significant risk factors for urolithiasis. It mainly showed patients with MetS were found to have a significantly higher proportion of urolithiasis than those without MetS. Among the components of MetS, hyperglycemia and hypertension were identified as the major influencing factors for urolithiasis in this region and revealing a trend toward younger ages among patients with urolithiasis. Moreover, ethnic and sex factors were found to significantly affect the occurrence of urolithiasis, with a notable association between increased blood lipid metabolic indicators and urolithiasis in male patients. Metabolic syndrome or metabolic related indexes such as blood sugar, triglyceride and diastolic blood pressure have significant influence on the occurrence of urolithiasis in Han patients. It is recommended that patients with MetS closely monitor the occurrence of urolithiasis, and clinical practices should incorporate urolithiasis screening.

## Author contributions

**Conceptualization:** Chuanjiang Ling.

**Data curation:** Renxiong Cao.

**Formal analysis:** Ge Zhang.

**Funding acquisition:** Chuanjiang Ling.

**Investigation:** Ge Zhang.

**Methodology:** Ge Zhang.

**Project administration:** Chuanjiang Ling.

**Resources:** Qiang Yao.

**Software:** Renxiong Cao.

**Supervision:** Zhipeng Liu.

**Visualization:** Chang Liu.

**Writing – original draft:** Ge Zhang.

**Writing – review & editing:** Chuanjiang Ling.
